# Mitigation of Methotrexate-Induced Intestinal Mucositis in Male Wistar Rats by Gallic Acid: The Role of HGF and C-Met Genes

**DOI:** 10.1155/jt/9990692

**Published:** 2025-03-17

**Authors:** Reza Norouzirad, Khashayar Zahedi, Mohammad Mehdi Behvandi, Abbas Moridnia, Susan Sabbagh

**Affiliations:** ^1^Department of Biochemistry, School of Medicine, Dezful University of Medical Sciences, Dezful, Iran; ^2^Student Research Committee, Dezful University of Medical Sciences, Dezful, Iran; ^3^Department of Genetics and Molecular Medicine, School of Medicine, Dezful University of Medical Sciences, Dezful, Iran; ^4^Infectious and Tropical Diseases Research Center, Dezful University of Medical Sciences, Dezful, Iran; ^5^Department of Anatomical Science, School of Medicine, Dezful University of Medical Sciences, Dezful, Iran

**Keywords:** gallic acid, HGF and C-met gene expression, IL-1β, intestinal mucositis, methotrexate, TNF-α

## Abstract

**Purpose:** Gastrointestinal mucositis (GI-M) is the most common adverse effect of methotrexate (MTX). Gallic acid (GA) is a polyphenolic component rich in green tea, gall nuts, hops, grapes, and oak bark and has anti-inflammatory and antioxidant properties. The aim was to investigate the impact of GA on proinflammatory cytokines, expression level of hepatocyte growth factor (HGF) and C-met genes, and histopathological alterations of MTX-induced GI-M in rats.

**Methods:** Twenty-four male Wistar rats were randomly divided into four groups: control, GA, MTX, and MTX + GA. Mucositis was induced in the experimental groups (MTX and MTX + GA) through three intradermal injections (the third to fifth days) of 2.5 mg/kg MTX in the suprascapular region. The GA group received 100 mg/kg GA via gavage, while the control group received normal saline by gavage (7 continuous days) and via intradermal injection (the third to fifth days) in the suprascapular region. The intestinal jejunal tissue and serum were analyzed for HGF and C-met mRNA expression, as well as levels of tumor necrosis factor-α (TNF-α) and interleukin-1 β (IL-1β). In addition, a histopathological study was to eperformedvaluate the villi of mucosa and fibrosis of submucosal layers.

**Results:** Decreased levels of HGF and C-met gene expression in the MTX group were significantly increased by GA administration (*p* < 0.05). GA administration decreased the elevated levels of TNF-α and IL-1β (*p* < 0.001) in the MTX group. Histopathological findings showed an adverse effect of MTX in mucosa which was relatively ameliorated in the MTX + GA ones.

**Conclusion:** GA could increase HGF and C-met expression, decrease inflammatory cytokines, and improve histological injuries, affected by MTX, indicating a beneficial role for GA following GI-M.

## 1. Introduction

Methotrexate (MTX) is a type of chemotherapy drug. The use of MTX in both adult and pediatric patients involves either as a standalone treatment or in combination with other medications. MTX disrupts folate synthesis and halts cell division by directly inhibiting DNA synthesis. Although this aspect of the MTX mechanism is effective in inhibiting rapidly dividing cancer cells, it can also damage the normal function of organs with high rates of cell division, such as the epithelial cells lining the intestinal tract [1]. Like other cytotoxic compounds, MTX results in the destruction of the gut epithelial barrier and an increased inflammatory response, which can ultimately cause gastrointestinal mucositis (GI-M). GI-M is indicated by damage to the mucosa lining and the submucosal layer [2]. While oral mucositis is studied in detail, maybe because of easy access to the oral cavity, research on GI-M remains sparse [3].

Patients on medication related to GI-M display a wide range of symptoms of the gastrointestinal tract, including diarrhea, nausea, vomiting, abdominal pain, and infections. Even with decades of clinical and experimental studies, GI-M continues as an unsolved medical problem, and it is widely agreed upon that implementation of effective strategies is crucial in the prevention and treatment of GI-M, so that conventional treatments for intestinal mucositis, including loperamide and zinc derivatives and mesalazine are not very effective [4]; therefore, it seems that no single treatment could have a significant impact on clinical management [5–7]. Currently, GI-M development involves five interactive and overlapping stages: (A) Direct cytotoxicity (i.e., irreversible damage to DNA and reactive oxygen species [ROS] production) that activates signaling factors such as caspase-1/3, Bcl-2, Wnt/β-catenin, nuclear factor-kappa B (NF-кB), and their associated pathways; (B) stimulation of messenger molecules leading to downstream immune signaling and the release of proinflammatory cytokines such as tumor necrosis factor (TNF), interleukin 6 (IL-6), and IL-1 beta (IL-1β); (C) strengthening of messenger molecules that worsen the injury of the organ; (D) ulceration and breakdown of the intestinal epithelium; and (E) unprompted healing characterized by cell proliferation [8].

Hepatocyte growth factor (HGF) is synthesized in stromal and mesenchymal cells. HGF helps epithelial cells to proliferate, stimulates the formation of new epithelial cells, morphogenesis, and undergoes angiogenesis in various organs through tyrosine phosphorylation of the cognate receptor, Met (the HGF receptor) [9]. In addition, several studies have shown that HGF can suppress common proinflammatory pathways and reduce acute and chronic inflammation in various diseases (e.g., inflammatory bowel disease [IBD], airway inflammation, and glomerulonephritis) [10–12]. Met, the HGF receptor, has been characterized as a C-met proto-oncogene, which catalyzes the production of a transmembrane receptor tyrosine kinase [13]. Upon binding to the Met subunit, HGF triggers the phosphorylation of the Met subunit, leading to the recruitment of intracellular signaling molecules [14], that induce several biological responses including proliferation, cell survival, morphogenesis, and angiogenesis. According to Xian et al., in a rat model of MTX-induced GI-M, upregulation of HGF and C-met expression facilitated intestinal mucosal recovery [15].

Barbosa Oliveira et al. highlighted that, in addition to the direct cell damage caused by chemotherapy in GI-M, oxidative stress mediators are also released. These mediators, in turn, induce the expression of proinflammatory cytokines, such as IL-1β and TNF-alpha (TNF-α), promoting the damage and rupture of the intestinal epithelial barrier [16]. Microscopic characterization of intestinal damage includes villi fusion, loss of crypt architecture with cyst formation, inflammatory cell infiltration, and submucosal layer fibrosis [17–19].

One of the main signs of many malignancies has been identified as malnutrition. Weight loss and diarrhea are frequent signs of malnutrition seen in cancer patients, particularly when the disease is advanced [20]. An increasing number of studies indicate that a nutritious, well-rounded diet can lower the likelihood of developing cancer and enhance the effectiveness of cancer therapies [21]. Indeed, individuals with cancer may require adjunctive therapies, such as dietary and lifestyle modifications to improve their chances of survival and manage their illness more effectively [22]. The major metabolites in plants are phenolic compounds [23]. More than 8000 natural phenolic compounds have been identified [24]. A diet enriched in phenolic compounds exhibits potent anticancer activities to combat various diseases associated with oxidative stress [25], by antioxidant and anti-inflammatory activities [26].

Gallic acid (GA) (3,4,5-trihydroxy benzoic acid) is a phenolic compound widely found in green tea, gall nuts, hops, red wine, grapes, and oak bark [27]. It is widely acknowledged from the literature that GA owns several pharmacological properties, as well as antitumor, antimutagenic, anti-inflammatory, antifibrosis, and antioxidant activities [27–29].

The present study aimed to evaluate the probable protective role of GA on

i. Tissue expression levels of HGF and C-met in the rat model of MTX-induced GI-M.
ii. Serum levels of proinflammatory cytokines of TNF-α and IL-1β.
iii. Histopathological conditions by villus examination and amounts of collagen fibers in the mucosa and submucosal layers.

## 2. Materials and Methods

### 2.1. Animals

Male Wistar healthy rats (24 rats), in the weight range of 190–210 gr, were purchased from Ahvaz Animal Laboratory, Ahvaz, Iran. The rats were kept in standard polypropylene cages (three rats per cage), in a controlled environment with a 12/12-h light–dark cycle, temperature of 23 ± 2°C, and humidity of 50 ± 5%. They were fed with a standard normal pellet diet (Pars Animal Feed Co.), received water ad libitum, and were acclimated for 1 week in the laboratory before the intervention.

All study procedures and animal care were approved by the Ethical Committee of Dezful University of Medical Sciences (DUMS), Dezful, Iran (Ethic code: IR.SBMU.Endocrine.Rec.1396.399) and finally, all of the animal experiments were reported following ARRIVE guidelines [30].

### 2.2. Experimental Design

Rats were randomly divided into four control (C), MTX, GA, and MTX + GA groups of 6 rats each. As seen in Diagram no.1, interventions started from Day 1 to Day 7. The rats in the control group were treated with 0.6 mL normal saline via gavage for 7 consecutive days, and rats of the GA and MTX groups were treated with 100 mg/kg of GA (Sigma-Aldrich, G7384) via gavage from Day 1 to Day 7 [31] consecutively. Furthermore, the animals of the MTX group were treated with 2.5 mg/kg of MTX (Sigma-Aldrich, CAS no. 133073-73-1) via subcutaneous injection in the suprascapular region for three consecutive days, the third to fifth days, of the study [15, 32].

During the treatment time, we comply with the protocol of animal experimental guidelines of the National Institutes of Health guide for the care and use of Laboratory animals (NIH Publications no. 8023, revised 1978).

**Figure 1:**
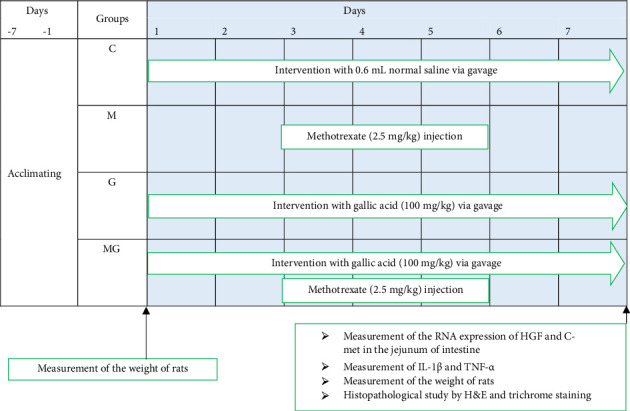
The summary of the experimental design.

Rat's body weight (using the Tefal scale, sensitivity: 0.1 g) was recorded before and last day of the intervention. At the end of the study, the rats were fasted for 12 h and were anesthetized using an intraperitoneal injection of xylazine (10 mg/kg) combined with ketamine (60 mg/kg). Then, a blood sample was obtained from the tail vein; after blood clot formation, the samples were centrifuged (10 min, 4°C, 5000 g) to separate sera, and stored at −80°C until measurement of TNF-α and IL-1β. Furthermore, the initial part of the intestinal jejunum was removed and divided into two parts, one part fixed in 10% formaldehyde for histological analyses, and the other part was stored at −80°C until further measurement of mRNA expression of HGF and C-met. (see Figure 1)

### 2.3. Biochemical Measurements

According to the kit protocol, serum TNF-α and IL-1β were measured using rat ELISA kits (IBL Company, Cat. nos. 27,194 and 27,193, respectively) [33]. All intra/interassay CVs of the TNF-α and IL-1β were < 6%.

### 2.4. Evaluation of Intestinal Jejunum Tissue

The initial part of the small intestinal jejunum was divided into two parts and allocated for histology [34] and gene expression [35].

#### 2.4.1. Measurement of mRNA Expression of HGF and C-met

mRNA extraction, cDNA synthesis, and real-time PCR have been described in detail elsewhere [35]. Intestinal jejunum tissue RNA was extracted by RNX-Plus kit (CinnaGen Co., Tehran, Iran), and cDNA was synthesized using the SinaClon First Strand cDNA synthesis kit (Cat. RT5201). Amplifications were performed using the SYBR Green Master Mix Kit (BioFact, South Korea) in an instrument (ABI Step One Plus, Applied Biosystems, Foster City, California, United States of America). A Housekeeping gene (GAPDH) was used to normalize target genes. The 2^^-ΔΔCt^ method was performed to calculate the relative mRNA expression levels (fold change) of HGF and C-met genes. The HGF, C-met, and GAPDH primer sequences are shown in Table 1.

#### 2.4.2. Histopathological Study

Tissue specimens treated with formalin fixative and paraffin embedding were used to prepare 5-μm-thick sections, deparaffinized with xylene, and then rehydrated with different grades of alcohol and histopathologically stained by hematoxylin–eosin (H&E) and Masson's trichrome staining [34]. Using light microscopy (Olympus BX52, Tokyo, Japan), all sections for the parameter's villus structure, probable presence of ulceration, amount of collagen fibers in the mucosa, and submucosal layers were evaluated.

### 2.5. Statistical Analysis

Using GraphPad Prism V.8.00, the statistical analysis of the data was reported as mean ± standard error of the mean (SEM). Body weight and biochemical measurements were analyzed by one-way ANOVA and Fisher LSD post hoc. The fold changes in mRNA expression of the two independent groups were compared by the independent *t*-test and Mann–Whitney *U* test. *p* < 0.05 was determined as statistically significant.

## 3. Results

### 3.1. Effects of MTX and GA on Rat's Body Weight

**Figure 2:**
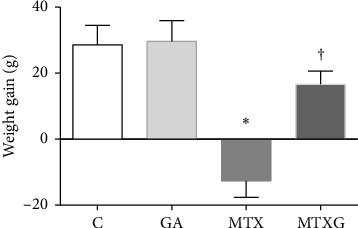
Effects of GA on body weight gain in C, GA, MTX, and MTX + GA groups (*n* = 6/group). The comparisons were performed by one-way ANOVA. *p* < 0.05 was considered statistically significant. ⁣^∗^ and ^†^ indicate significant differences compared to the control and MTX groups, respectively. C: control; GA: gallic acid; MTX: methotrexate; MTX + GA: methotrexate + gallic acid.

The general conditions of all treated rat groups were observed daily. The average body weights of control, GA, MTX, and MTX + GA on the first day were 198.0, 194.0, 197.7, and 198.0 g, respectively, which were not significantly different.  Figure 2 shows the body weight gain of rats on the first and last day of the study in each group. Rats in the MTX group experienced diarrhea. MTX decreased the body weight gain of MTX-treated rats (−12.7 + 5.0) compared to Control 1 (28.7 + 5.8) (*p*=0.0001). GA reversed the weight loss induced by MTX.

### 3.2. Effects of MTX and GA on TNF-α and IL-1β Concentrations

**Figure 3:**
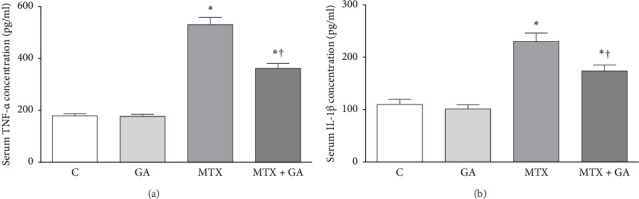
The impact of gallic acid on the serum levels of TNF-α (a) and IL-1β (b) in control and methotrexate rats. The comparisons were performed by one-way ANOVA. *p* < 0.05 was considered statistically significant. ⁣^∗^ and ^†^indicate significant differences compared to the control and MTX groups, respectively. The values are presented as mean ± SEM with a sample size of 6 per group. C: control; GA: gallic acid; MTX: methotrexate.

As shown in Figure 3(a), MTX increased TNF-α levels in the MTX group (293%, *p* < 0.001) than the control. GA decreased the levels of TNF-α in the MTX + GA group (69%, *p* < 0.001) compared to the MTX group.

As shown in Figure 3(b), MTX increased IL-1β levels in the MTX group (208%, *p* < 0.001) than the control. GA decreased the levels of IL-1β in the MTX + GA group (76%, *p* < 0.05) compared to the MTX group.

Although GA declined the increased levels of TNF-α and IL-1β by MTX, this decline did not reach the level of the control group.

### 3.3. Effects of MTX and GA on HGF and C-met mRNA Expression

**Figure 4:**
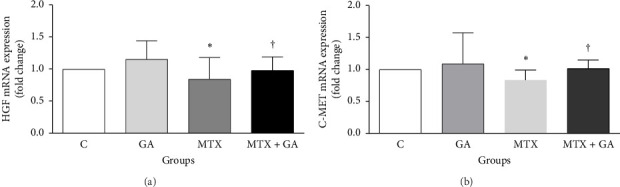
The impact of gallic acid on the expression of HGF (a) and C-met (b) genes in the jejunum of the small intestine. The comparisons were performed by one-way ANOVA. *p* < 0.05 was considered statistically significant. ⁣^∗^ and ^†^indicate significant differences compared to the control and methotrexate groups, respectively. The values are presented as mean ± SEM with a sample size of 6 per group. C: control; GA: gallic acid; MTX: methotrexate; MTX + GA: methotrexate and gallic acid.

As shown in Figure 4(a), MTX decreased the mRNA expression of HGF in the MTX group (15.5%, *p* < 0.05) compared to the control. GA increased the mRNA expression of HGF in the MTX + GA group by 13.6% (*p* < 0.05) compared to the MTX group, reaching the levels of the control group.

As shown in Figure 4(b), MTX decreased the mRNA expression of C-met in the MTX group (16.0%, *p* < 0.05) than control. GA increased the mRNA expression level of C-met in the MTX + GA group by 17.8% (*p* < 0.05) than the MTX group, reaching the levels of the control group.

### 3.4. Effects of MTX and GA on Histological Analyses of Intestinal Jejunum by H&E and Masson's Trichrome Staining

#### 3.4.1. Histopathological Results of Control and Other Groups Stained by H&E

**Figure 5:**
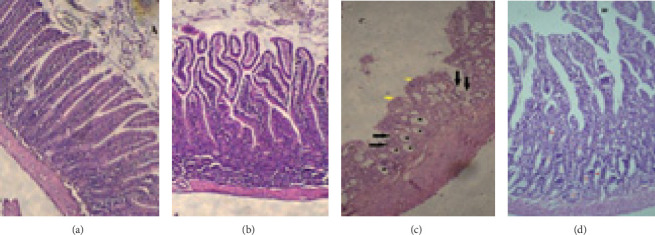
Photomicrographs of the jejunal sections stained with hematoxylin and eosin (× 100 magnification). (a) Normal jejunal tissue of the control group, notice finger-like villi, covered with simple columnar epithelium and intestinal crypts. (b) A nearly normal structure of the jejunal tissue in the gallic acid group. (c) Methotrexate group exhibits surface villus epithelial damage with villi fusion, flattening of surface epithelium (indicated by yellow arrows), ulceration of epithelium (black arrows), mucosal crypt damage, and cystic formation (black stars). (d) Jejunal tissue from the gallic acid + methotrexate group, highlighting tall villi covered with simple columnar epithelium and crypts in the lamina propria.

The prepared photomicrographs of H&E stained sections of jejunal tissues were evaluated.  Figure 5(a) shows the normal architecture of jejuna so that the villi appeared as finger-like projections that were covered by a single layer of columnar epithelial cells with oval basal nuclei; also, the cross-sections of crypts were seen in lamina propria.

Figure 5(b) shows the cross-section of jejunal tissue treated with GA so that the long slender villi with columnar epithelium, a normal appearance of crypts in the lamina propria, was seen.

Examination of the MTX group revealed loss of villus architecture, the fusion of villi, disruption and shedding of surface epithelium, and the presence of ulceration, flattening of enterocytes, atrophy of crypts, and development of cysts (Figure 5(c)). Pretreatment with GA could decrease tissue damage so that almost normal structure of villus, reconstruction of crypts, and healing of ulcers with normal appearance of the epithelium were shown (Figure 5(d)).

#### 3.4.2. The Results of MTX and Other Group Slides Stained by Mason's Trichrome

**Figure 6:**
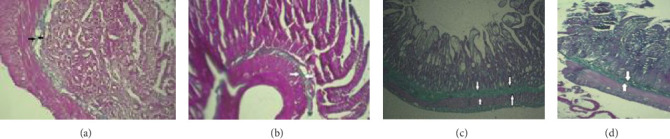
Photomicrographs of the jejunum tissue stained with Masson's trichrome (× 100 magnification). (a) The control group, where a delicate amount of green collagen fibers is observed in the submucosal layer. (b) A cross-section of the jejunum in the gallic acid group, with the amount of collagen fibers in the submucosal layer similar to that of the control group. (c) A thick layer of collagen bundles in the submucosal layer of the methotrexate group. (d) A decreased amount of green collagen bundles in the gallic acid + methotrexate group.

Light microscopic examination of control and GA groups stained by Masson's trichrome showed a minimal amount of green-collagen fibers in the lamina propria and submucosal layer (Figures 6(a) and 6(b)), while thick bundles of collagen fibers in the submucosa of the MTX group was revealed (Figure 6(c)). Finally, the examination of light microscopic slides of the MTX + GA group showed a reduced amount of green-colored collagen fibers in the submucosa, among the crypts (Figure 6(d)).

## 4. Discussion

Clinical symptoms of MTX-induced GI-M comprise mucosal ulcerations, diarrhea, weight loss [36], reduced appetite, and disturbed absorption [37]. In the present study, the administration of MTX caused diarrhea and significant weight loss. The pathobiology of GI-M initiates with the recognition of chemotherapeutic agents by submucosal blood vessels stimulating damage to the basal epithelial cells. Consequently, the injured cells activate stress mechanisms, leading to the release of proinflammatory cytokines and the generation of ROS [8]. In line with this, the released proinflammatory cytokines act as secondary messengers, activating mucosal-associated cells such as macrophages and endothelial cells, which in turn, release a cascade of proinflammatory cytokines, including TNF-α and IL-1β, and intensify tissue injury [38]. Our results revealed elevated serum TNF-α and IL-1β levels in rat models of MTX-induced GI-M compared to the corresponding control group.

Our study showed that MTX causes loss of intervillous spaces and fusion of villi, disruption of epithelial cells and ulceration, atrophy of crypts, and development of cysts in the jujena of the intestine (Figure 3(c)), which is consistent with the studies of Kaynar [39], de Araújo [17], and others [40]. These effects are due to the interaction between DNA content and the folic acid analog MTX. MTX binds to the dihydrofolate reductase enzyme (DHFR), inhibits DNA synthesis, and hinders the proliferation of the enterocytes in the small intestine's villi [41]. Moreover, most of the small intestine's villi were covered with flattened or cuboidal enterocytes (Figure 5(c)). This pathological observation could likely be attributed to the significant apoptosis of stem cells in the villus and the antimitotic effect of MTX [42].

Our results showed fibrosis induced by MTX. Ashmawy et al. [41] and Dadhania et al. [43] assessed the submucosal layer of the jejunum in rats experiencing GI-M conditions. They proposed that MTX induces submucosal fibrosis and increases the density of collagen fibers compared to the corresponding control group. Examination of Masson's trichrome-stained intestinal tissue slides revealed delicate collagen fibers in the lamina propria and submucosal layer of the jejunum in the control groups. In line with other studies, evident thick bundles of collagen fibers and distinct fibrosis were observed in the submucosal layer of the small intestine in MTX-treated rats (Figure 6(c)). The probable cause of fibrosis could be either the direct toxic effect of MTX or a consequential effect of inflammation [15].

The current study's results unveiled a notable decrease in the mRNA expression levels of HGF and its receptor, C-met, in the MTX group. This decline was coupled with an increase in proinflammatory cytokines TNF-α and IL-1β, alongside a sharp rise in tissue injury. Previously, Xian et al. reported similar findings regarding the reduction of the gene expressions in rat models of MTX-induced GI-M and concluded that the HGF gene has a direct effect on villi structure [15].

While the alternatives for preventing and treating chemotherapy-induced GI-M remain limited, insufficient energy and nutrient intake should prompt various nutritional interventions [37]. There is a lack of clinical evidence regarding nutritional outcomes for patients with mucositis. Nutritional intervention strategies are needed to address the deficiencies [37]. Some review studies have emphasized that treating patients with GI-M may be ineffective due to the lack of effective nutritional therapeutic strategies [44, 45].

GA inhibits the release of inflammatory factors (TNF-α and IL-1β/6) and other inflammatory mediators such as COX-2 and nitric oxide (NO), thereby reducing the inflammatory response [46]; it may act through the suppression of the NF-kB activation [28]. Our study yielded similar results with decreased expression levels of these inflammatory mediators and increased expression levels of HGF and its receptor, C-met, in GA-treated rats that experienced GI-M conditions. Histopathological findings showed improvement not only in the epithelial growth and crypt formation in villous ulcers but also in the reduction of cyst formation and fibrosis in the submucosa layer. Previous research by El-Lakkany et al. demonstrated the antifibrotic effect of GA in various liver conditions *in vivo* and *in vitro* [47], but there are limited studies on the effect of GA on epithelial cell repair [48]. A large body of evidence shows that HGF has remarkable anti-inflammatory and antifibrotic potential and could ameliorate histopathological lesions in different organs such as the kidney, liver, lung, and so on [49–51]. The possible mechanism of treatment of mucositis in the present study by GA may be exerted through the increase of HGF expression, although its direct role in the healing of mucositis is also discussed. Indeed, it is speculated that GA can increase the expression of HGF by reducing oxidative stress under its intrinsic antioxidant property.

As a limitation, we could not investigate the effect of GA on female rats. Also, other signaling pathways specifying the exact mechanism of action of GA can be performed. However, the present study showed the harmful effects of MTX and the protective role of GA with strong evidence using histology (by trichrome and H&E staining), which provides first-hand information, along with molecular and biochemical examinations.

## 5. Conclusion

It is concluded that GA intervention can ameliorate MTX-induced increased levels of proinflammatory cytokines, tissue damage, and fibrosis. The possible mechanism of protective effects of GA could be via modulation of inflammation and oxidative stress, which culminate in to increase in the expression of HGF, followed by repairing of intestinal mucositis. Therefore, GA can be considered an adjunctive treatment for MTX-induced intestinal mucositis.

It can be recommended to consider the potential of using GA as an adjunctive treatment with MTX therapy. However, before using GA with a medicinal dose, it is necessary to explore its efficacy and safety in clinical trial studies.

## Figures and Tables

**Table 1:** Primers used in real-time PCR amplification.

Gene name	GeneBank accession No.	Primer sequence	Product length (bp)
HGF	24,446	F. 5′ATATGGAGGATTTACACCGTC3′	21
		R. 5′TGTAGCACCAAGGTCCATG3′	19

C-met	24,553	F. 5′GAGCACTGTTTCAATAGGACC3′	21
		R. 5′CGTTAAGAGTACATGGTTGAGC3′	22

CAPDH	24,383	F. 5′CTCATGCGACTTCAACAGC3′	19
		R. 5′CGTCTACATTGTCATACCAGG3′	21

## Data Availability

The data used to support the findings of this study are available from the corresponding author upon reasonable request.
